# On-water Rowing Biomechanical Assessment: A Systematic Scoping Review

**DOI:** 10.1186/s40798-024-00760-2

**Published:** 2024-09-27

**Authors:** Natalie Legge, Conny Draper, Katie Slattery, Damien O’Meara, Mark Watsford

**Affiliations:** 1 School of Sport, Exercise and Rehabilitation, Faculty of Health, University of Technology Sydney (UTS), Sydney, NSW Australia; 2 Human Performance Research Centre, University of Technology Sydney (UTS), Sydney, Australia; 3 Lausanne, Switzerland; 4 NSW Institute of Sport, Sydney, NSW Australia

**Keywords:** Rowing, Biomechanics, Technique, Performance, On-water environment

## Abstract

**Background:**

Biomechanical parameters can distinguish a skilled rower from a less skilled rower and can provide coaches with meaningful feedback and objective evidence to inform coaching practices on rowing technique. Therefore, it is critical to understand which technical characteristics can be related to the fundamental rowing performance indicators. The aim of this systematic scoping review was to describe the current focus and density of rowing biomechanics research specific to on-water rowing and provide a guide for practitioners and researchers on future directions for on-water rowing biomechanics research.

**Methods:**

All peer-reviewed publications involving the on-water assessment of rowing biomechanics were reviewed from four databases (SPORTDiscus, PubMed, Sage online journals, and Web of Science). Search results returned 1659 records, of which 27 studies met the inclusion criteria for the review.

**Results:**

All reported variables were collated and summarised according to the three main measurements of basic mechanics: time, space and force. Study characteristics were collated to provide a descriptive overview of the literature. The main categorical variables included time, distance, velocity, acceleration, force, power and crew synchrony.

**Conclusion:**

Data extraction revealed gate force, horizontal oar angle and boat velocity as the most reported variables with numerous subcategories of metrics within each measure. A framework to help guide and standardise on-water rowing biomechanical assessment and the establishment of standards for environmental data collection could help guide practitioners and researchers in the on-water rowing environment. This scoping review was registered on the Open Science Framework (https://osf.io/8q5vw/).

## Key Points

- While the literature has reported on an extensive range of biomechanical metrics relevant to rowing performance, however, the variability of reported measures across the different boat classes, sex and skill levels makes the collation of data challenging.
- The rate at which a rower can apply force and the ability to maintain the force into the finish are distinguishing features of elite rowing while prioritizing the measurement and application of power is essential for effectively monitoring and controlling training loads, as well as for refining technique.
- The development of a standardized framework for on-water rowing biomechanical assessment, coupled with established protocols for environmental data collection, would provide practitioners and researchers with a structured approach for navigating the on-water rowing context.

## Background

Attributes of rowing performance incorporate all facets of the athlete including physiology, psychology, biomechanics and technique [1]. The physical attributes of power, strength, anaerobic and aerobic capacity are critical and an effective transfer of these qualities from the rower to the boat is essential for optimal rowing performance [1]. Furthermore, poor rowing technique can be detrimental to performance and increase the risk of injury [2]. The main performance indicator of rowing is race time over 2000 m. Consequently, boat velocity and propulsive force are also closely associated with performance [3–6]. Rowing biomechanics research has attempted to identify technique characteristics of successful rowing; however, it is unclear which characteristics can be related to the fundamental performance indicators [4]. On-water rowing research is challenging due to the logistical difficulty in controlling the environmental conditions [6, 7] and as a result, much of the biomechanical rowing research has been conducted on rowing ergometers in laboratory settings [8, 9]. However, biomechanical instrumentation systems for the rowing boat are becoming more accessible, reliable, and valid for practitioners and researchers to transition more research and technical assessment out of the laboratory and into the rowing boat [6, 10, 11].

There are two types of rowing: sculling and sweep rowing. Sculling involves two oars, with an oar handle in each hand, whilst in sweep rowing each person only has one oar with both hands gripping the same oar handle [12]. In addition, there are a range of boat categories within each type of rowing. Sculling includes the single (1x), double (2x) and quadruple scull (4x) whilst sweep rowing includes the pair (2− ), four (4− ) and coxed eight (8 +). The pair, four and quadruple scull can be coxless or coxed which refers to the addition of a coxswain to steer the boat and motivate the crew during the race [12]. Lastly, there are two weight divisions: lightweight and heavyweight for both men and women. Lightweight men and women are required to have a crew average for body mass of 70 kg and 57 kg respectively although this will no longer be contested at the Olympic level following Paris 2024. These classifications are unique to rowing and increase the variability of reported outcomes in the rowing biomechanics literature. Therefore, depending on the participant characteristics and demographics within each study, it is difficult to collate and compare results across previous studies.

Technique in relation to performance is often evaluated and taught subjectively by the coach using their experience and innate ability to observe and provide verbal feedback [13]. It is measured less frequently by objective measures of biomechanical assessment [4]. Performance in its simplest form can be measured by race results and boat speed; however, performance level can also be defined by an evaluation of skill and technique against a standard set of biomechanical criteria [14]. In such scenarios, the complexity is in establishing the benchmark parameters. Research focussed on biomechanical parameters to distinguish a skilled rower from a less skilled rower can provide coaches with more meaningful feedback and objective evidence to inform coaching practices on rowing technique [3]. Boat mechanics and body kinematics continue to be areas of research interest in rowing due to their implications for both performance outcomes and injury risk [15]. Where available, this information can support and inform the coach, athlete and support staff when assessing and refining technique for improving on-water performance.

Rowing in training, testing and racing environments is affected by weather conditions; specifically, wind direction and speed, and water temperature [7] and the somewhat limited research can be partly attributed to the logistical difficulties and environment variability experienced during on-water rowing [6]. Certain environmental aspects can be managed through using enclosed waterways with no tidal flow, monitoring wind and water temperature, and conducting testing sessions on a buoyed racecourse. Further, kinematic rowing research is limited in the on-water environment due to the reliance on video digitization to assess joint position and movement. Inertial sensors are emerging as devices that can precisely assess various biomechanical aspects of rowing. However, the literature is currently lacking guidelines on methodology and appropriate analysis in the on-water environment [16].

Research in rowing has narratively summarised fundamental principles relevant to improving performance, such as maximising the propulsive impulse and minimising drag impulse on the system [3]. Extensive review of force application profiles has been reported, but a lack of experimental research exploring the stretcher forces has been highlighted [17]. Recommendations suggest that ideal profiles of force should be investigated, including the stretcher forces, to determine if there is an optimal interval of sequencing between the gate and stretcher throughout the stroke cycle [4]. Differences between ergometer rowing and on-water rowing continue to be a point of interest, but due to the convenience of the laboratory setting, ergometer research continues to dominate the literature [6, 18]. The assessment of joint position and body segment coordination for rowing has predominantly been undertaken on instrumented rowing ergometers, due to the availability of accurate motion tracking equipment in a laboratory setting [2, 8]. Criterion-standard motion analysis systems can provide reliable and accurate information on body kinematics [19, 20]; however, on-water instrumentation systems remain limited in this area and have the additional difficulty of variable environmental conditions [21]. Measures of rowing performance have been reviewed, although not specific to on-water assessment, with a summarised account based on the validity and reliability of known systems and devices [6]. Despite conclusive statements in published research predicting that on-water performance measures may eventually surpass ergometer measures, over the past 20 years ergometer measures for rowing performance have continued to outpace on-water assessment options [6]. Systematic reviews are increasingly popular in rowing; however, multiple disciplines have been included in the same reviews including biomechanics, physiology, hydrodynamics and electromyography. This has led to summaries that are non-specific and arguably too broad [8, 9]. In contrast, this scoping review addresses this gap by focussing exclusively on the on-water rowing literature. A systematic scoping review is appropriate for this topic as it presents an overview of a diverse body of rowing biomechanics literature. The aim of this systematic scoping review was to describe the current focus and density of rowing biomechanics research specific to on-water rowing and provide a guide for practitioners and researchers on future directions for on-water rowing biomechanics research.

## Methods

### Design and Search Strategy

This scoping review was completed according to the Preferred Reporting Items for Systematic Reviews and Meta-Analyses extension for Scoping Reviews (PRISMA-ScR) [22]. A systematic search of the literature involving biomechanical variables associated with rowing performance was conducted using four online databases to perform the electronic search: SPORTDiscus, PubMed, Sage online journals, and Web of Science. A search strategy was developed to identify all relevant studies related to rowing biomechanics and performance. Systematic searches were conducted in each database. All databases were initially searched from the earliest record up to and including April 2020. The search was updated with new results from all databases to include up to 29 September 2023. The search strategy combined terms following the Population, Concept, Context (PCC) framework with a full list of terms in Table 1 [23]. The term “on-water” was not specified in the search strategy and “ergometer” was not an excluded term as part of the search strategy. This search strategy allowed for an assessment of the rowing biomechanics literature on the ratio of ergometer and on-water rowing studies. This review protocol was registered with Open Science Framework (https://osf.io/8q5vw/).

**Table 1:** Search term strategy

Population	Title or Abstract: ‘rowing OR rower’	
Concept	(All text)—(biomechanics OR kinetic OR kinematic OR force OR velocity OR acceleration OR power OR stroke length)	AND
Context	(All text)—(performance OR “sport performance” OR technique OR skill OR “level of expertise”)	AND

### Study Selection

The database search was conducted by one author (NL) using the search strategy detailed in Table 1, and the search results were uploaded to the web-based screening software, Covidence (Veritas Health Information, Melbourne, VIC, Australia) for the screening process. Duplicates were automatically removed. The title and abstracts were screened by two reviewers (NL and CD) using the inclusion and exclusion criteria in Table 2. Any disagreements about study inclusion or exclusion that could not be resolved through discussion were decided by a third author (MW). After the title and abstract screening, all articles for full text screening were retrieved and assessed by two authors (NL and CD) using the same inclusion and exclusion criteria. Reference lists from full text studies and reviews were also screened for potentially relevant articles to be included in the full text screening. Attempts were made to contact authors of select studies to request full text articles that were unavailable or to retrieve any missing relevant information. Studies were eligible for inclusion if they were assessing, examining, or exploring biomechanical variables that may have an association with on-water rowing performance.

**Table 2:** Inclusion and Exclusion Criteria

Inclusion criteria	Exclusion criteria
Published in English	Systematic reviews, meta-analyses, other review articles, conference proceedings
Publication from any year	Articles without an abstract and/or no full text available
Peer-reviewed Journal articles	Population: para-rowers, spinal cord injury or paraplegic participants
Study design: experimental, quasi-experimental, non-experimental, or observational	Study is investigating equipment, modelling simulation methods, motor learning or feedback methods
Study includes on-water rowing assessment in relation to rowing performance	Study utilises the rowing ergometer as the modality for assessment
Study involves observing, evaluating, or investigating some aspect of rowing biomechanics in relation to rowing performance	Validity and reliability studies on new equipment or systems

### Data Extraction

To generate an overview of the existing on-water rowing biomechanics literature, data were extracted pertaining to study details (duration, country), population (sample size, age, training level and status, performance level), instrumentation systems used, and specific variables reported. Extracted data were entered into a customised online spreadsheet allowing review by multiple authors. As scoping reviews do not necessarily synthesise all extracted data, a tabular summary has not been provided in this text. No risk of bias assessment was conducted due to this being a descriptive scoping review, and effects or prevalence were not reported.

## Results

### Study Characteristics

**Fig. 1:**
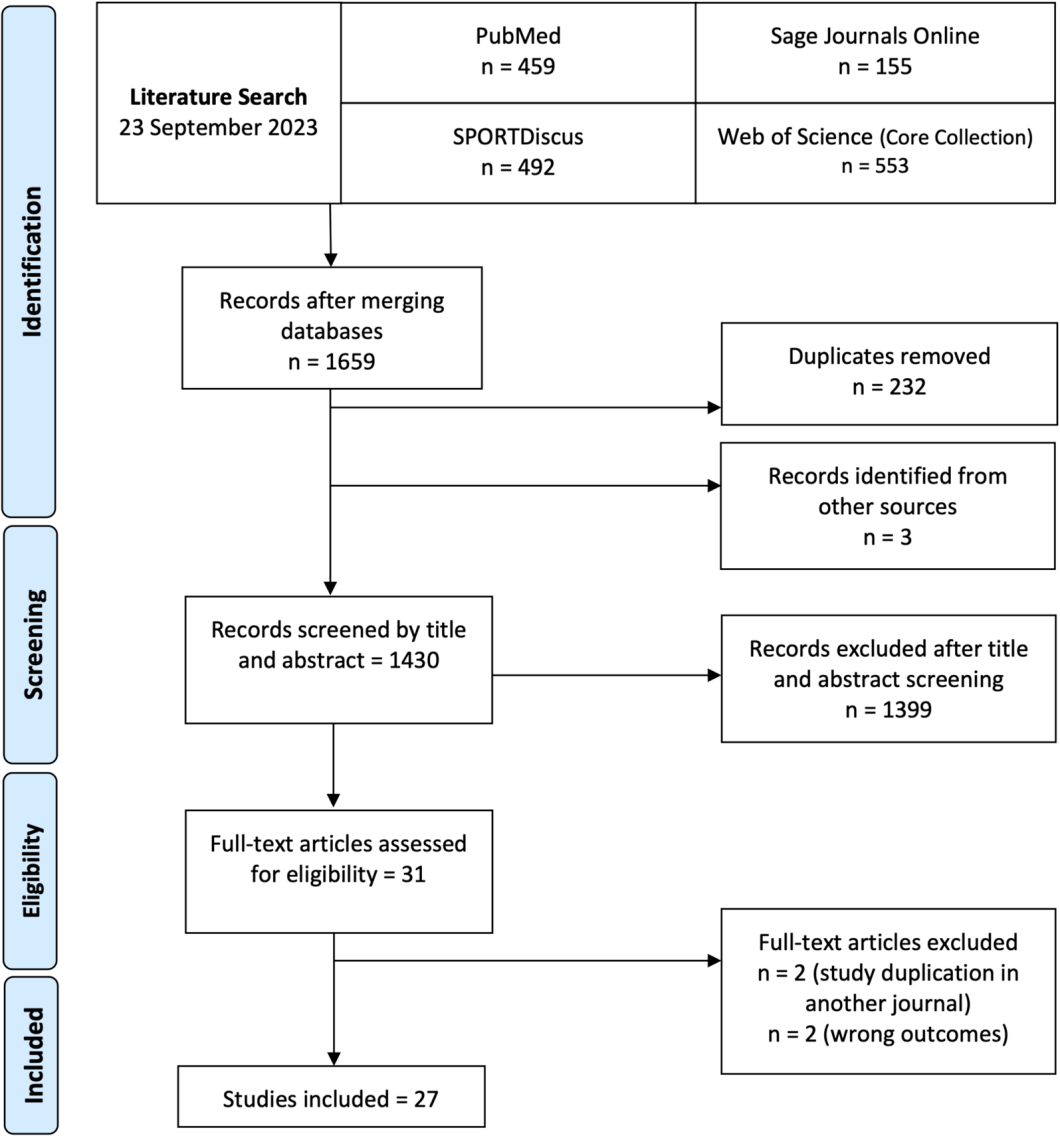
PRISMA flowchart of the literature search and screening process

**Fig. 2:**
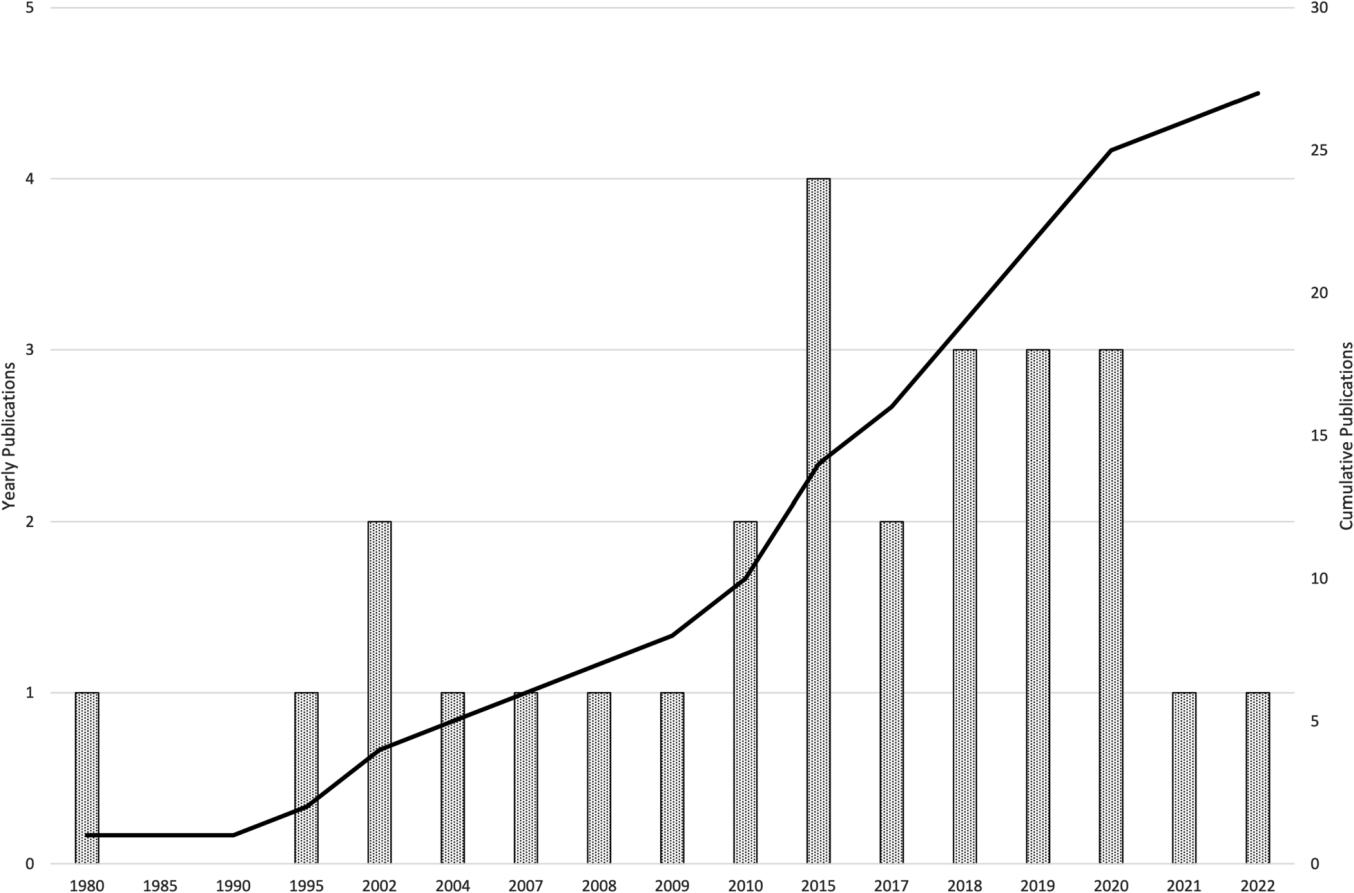
Number of on-water rowing biomechanics publications by year (Cumulative publications – black line)

From the initial 1430 articles that were screened by title and abstract, 31 articles were assessed by full text and 27 articles were subsequently included for review. The flow of articles from identification through to inclusion is presented in Fig. 1. Across the 27 studies, on-water biomechanical rowing testing was conducted in various boat classes ranging from single sculls to coxed eights. This included 11 studies using single sculls, 9 studies using coxless pairs, 1 study using double sculls, 2 studies using coxless fours and 4 studies using coxed eights. Small boat categories including the single for sculling and the coxless pair for sweep rowing were dominant across the literature reflecting an interest in individual rower output rather than the combination of a larger crew. The majority of the included studies in this scoping review were observational and cross-sectional in design. Ten of the 27 studies comprised only male participants, 3 studies involved only female participants and 13 studies included both male and females. One study did not define the participant demographics other than it was a group of elite and sub-elite rowers [5]. According to authorship, 10 nations have contributed to the peer-reviewed, on-water rowing biomechanical literature, with a slight increase in the number of publications since 2015 (Fig. 2). Both commercial and custom-built instrumentation systems have been utilised to measure the specific variables of interest to each study. The specifications of each system are beyond the scope of this review. However, details for the commercial systems can be found on the relevant websites, in particular, Peach Innovations, BioRowTel and Weba Sport. Table 3 summarises the study characteristics including author group, journal source, sample size, and participant demographics.

**Table 3:** Study Characteristics

References	Study Location	Journal	Instrumentation System	Sample Size	Age (years)	Age Category	Training Level / Status	Boat Class	Weight Category
Baudouin & Hawkins 2004 [47]	USA	J Biomech	Custom instrumented oar force and angle (foil strain gauges and linear potentiometer)	8M	Unknown	University age	Collegiate	2-	HW
Cuijpers et al. 2017 [45]	Netherlands	Scand J Med. Sci Sports	Custom instrumented oar force and angle (foil strain gauges and linear potentiometer)	24M, 3F	20 years ± 7	Experienced rowers	National	2x	HW & LW
Doyle et al. 2008 [28]	Australia	Impact of Technology on Sports II	Custom Oarlock 2D load transducers, Horizontal oar shaft potentiometer, Seat drum & reel transducer	28M	Unknown	Unknown	International	2-	HW & LW
Doyle et al. 2010 [14]	Australia	Sports Biomech	Custom Oarlock 2D load transducers, Horizontal oar shaft potentiometer, Seat drum & reel transducer	28M	22.8 ± 3.7	Underage and senior	National	2-	HW & LW
Gravenhorst et al. 2015 [35]	Switzerland, Australia	IJCSS	Minimaxx (accelerometer & gyroscope), Peach Innovations (gate force & gate angle)	4F	Unknown	Senior	International	2x	unknown
Hill 2002 [46]	Germany	J Sports Sci	Four strain gauges (HBM, Darmstadt, Germany)—glued onto Concept 2 macon bladed oars	20M	22–31	Senior	International	4-	LW
Hill & Fahrig 2009 [26]	Germany	Scand J Med Sci Sports	MMS2000 (FES, Berlin, Germany, Bohmert & Mattes 2003)	15M	17–31	Underage and senior	Club, National	2-	HW
Hofmijster et al. 2007 [65]	Netherlands, Australia	J Sports Sci	RowSys measurement & telemetry system (Uni Sydney & NSWIS—Smith & Loschner, 2002)	6M, 3F	19–26	Underage and senior	"Experienced" rowers (2-12yrs experience)	1x	HW
Kleshnev 2010 [38]	Australia, UK	J. Sports Eng. Technol	BioRowTel, Berkshire, GB	294 crews	Unknown	Unknown	National & International		HW & LW
Lintmeijer et al. 2018 [39]	Netherlands	J Sports Sci	Peach PowerLine instrumentation systems (Peach Innovations, UK)	5M, 4F	19–42	Underage, senior, masters	"Experienced" rowers (2-20yrs experience)	1x	HW & LW
Liu et al. 2020 [33]	China	Sports Biomech	Custom system built similar to ROWX system (Weba Sport)	10M	21.8–29.4	Senior	International	1x	HW
Martin & Bernfield 1980 [29]	USA	Med Sci Sports Exerc	Video analysis	8M	Unknown	Senior	International	8 +	HW
Mattes & Wolff 2019 [34]	Germany	Int J Perform Anal Sport	MMS2000 (FES, Berlin, Germany, Bohmert & Mattes 2003)	16M, 16F	Under 19	Juniors (under19)	International Junior	8 +	HW
Mattes et al. 2015 [72]	Germany	JHSE	MMS2000 (FES, Berlin, Germany, Bohmert & Mattes 2003)	156	Under 19	Juniors (under19)	International Junior	8 +	HW
Mattes et al. 2015 [32]	Germany	Int J Perform Anal Sport	MMS2000 (FES, Berlin, Germany, Bohmert & Mattes 2003)	24M	Under 23	Juniors, under 23	International Junior	4-	HW & LW
Mattes et al. 2019 [41]	Germany	Biol Exerc	MMS2000 (FES, Berlin, Germany, Bohmert & Mattes 2003)	12M	Unknown	Senior	International, National	1x	HW & LW
Millar et al. 2015 [18]	NZ	Sports	Peach PowerLine instrumentation systems (Peach Innovations, UK)	4M, 4F	19–24	Underage	National Junior	1x	HW & LW
Perić et al. 2019 [25]	Serbia	Int J Perform Anal Sport	BioRowTel, Berkshire, GB	12M	23–29	Senior	International, Collegiate	2x	HW
Smith & Loschner 2002 [5]	Australia	J Sports Sci	Rowsys2 system (custom integrated system)	Unknown	Unknown	Unknown	National, International	1x, 2-	HW & LW
Warmenhoven et al. 2017 [61]	Australia, Ireland	Scand J Med Sci Sports	Rowsys2 system (custom integrated system)	27F	25.6 + / 4.9	Senior	National, International	1x	HW & LW
Warmenhoven et al. 2018 [17]	Australia, Ireland	Scand J Med Sci Sports	Rowsys2 system (custom integrated system)	27F	25.6 + / 4.9	Underage and senior	National, International	1x	HW & LW
Warmenhoven et al. 2018 [44]	Australia, Ireland	JSAMS	Rowsys2 system (custom integrated system)	20M, 20F	25.6 + / 4.9	Underage and senior	National, International	1x	HW & LW
Wing & Woodburn 1995 [48]	UK	J Sports Sci	Custom instrumented oar force (metal foil strain gauges)	5M, 4F	19–24	University age	Club	8 +	HW
Holt et al. 2020 [30]	Australia	Front sports act Living	Peach PowerLine instrumentation systems (Peach Innovations, UK)	14M, 17F	18–24	Underage and senior	National Junior	1x, 2-	HW
Holt et al. 2021 [40]	Australia	PLoS One	Peach PowerLine instrumentation systems (Peach Innovations, UK)	23M, 21F	18–24	Underage and senior	National Junior	1x, 2-	HW
Holt et al. 2022 [31]	Australia	Scand J Med Sci Sports	Peach PowerLine instrumentation systems (Peach Innovations, UK)	14M, 16F	18–24	Underage and senior	National, International	1x, 2-	HW
Held et al. 2020 [42]	Germany	Eur J Sport Sci	BioRowTel, Berkshire, GB	69	18–22	Underage	Club—National	1x	HW

HW: heavyweight rowers; LW: lightweight rowers; M: male; F: female; USA: United States of America; UK: United Kingdom; NZ: New Zealand; 2D: two-dimensional; 1x: single scull, 2x: double scull, 2-: coxless pair, 4-: coxless four, 8 + : coxed eight

### Biomechanical Variables

**Fig. 3:**
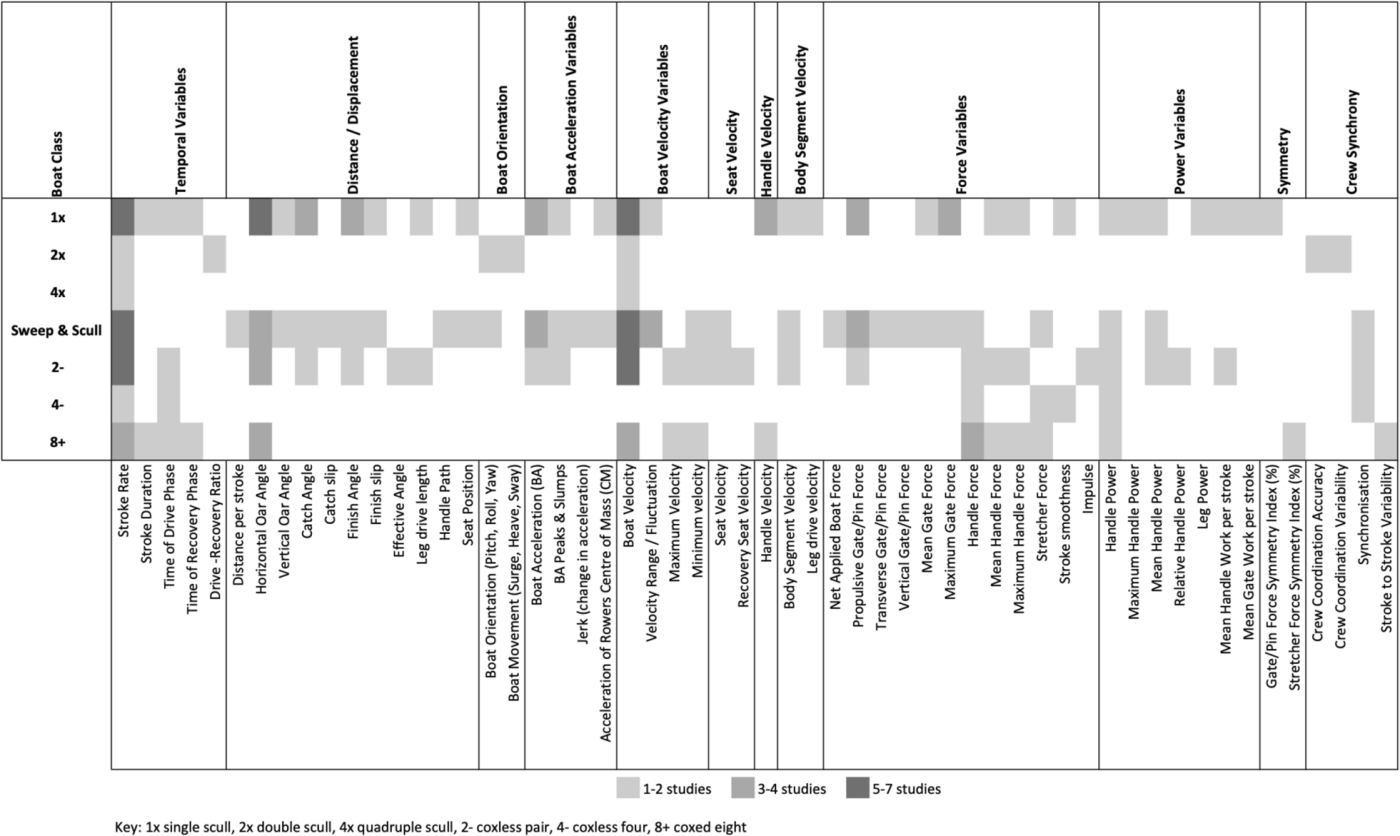
Heat Map of Biomechanical Variables Reported in the Literature for On-water Rowing

All reported variables in rowing are derived from one or a combination of the three main groups of basic mechanical measurements: time, space, and force [24]. The heat map in Fig. 3 visualizes the prevalence of various biomechanical variables reported by the 27 studies, categorizing them into domains including timing, oar angle, positioning, force, velocity, acceleration, power, and crew synchronization. The heat map arranges broader categories along the top fields with corresponding specific metrics detailed on the lower axis, accentuating the extensive range and diversity of biomechanical measurements in rowing research. This visualization underscores the widespread variability in the biomechanical metrics reported within the literature. Reported stroke rate ranged from 20 strokes per minute (spm) up to 41 spm. A number of studies used a step rate testing protocol [5, 25, 26], where a short distance, such as 250 m, is completed by crews and repeated over a series of increasing stroke rates, providing a spectrum of performance outputs as intensity increases. However, some studies only extracted 1 or 2 stroke rates for analysis and to address their research question [5, 17]. The second measurement group: space, includes length, distance and angles. Reported examples include stroke length, distance per stroke, and horizontal oar angle. The third measurement group: force, has been reported in up to two planes: horizontal and vertical, and measured in a variety of locations including the gate, pin, handle and foot stretcher. Holt et al. [27] describes differences in the force sensor location between the gate, pin and handle [27]. Moreover, velocity and acceleration are products of time and space, and the combination of time, space and force produces mechanical rowing power.

#### Velocity

**Fig. 4:**
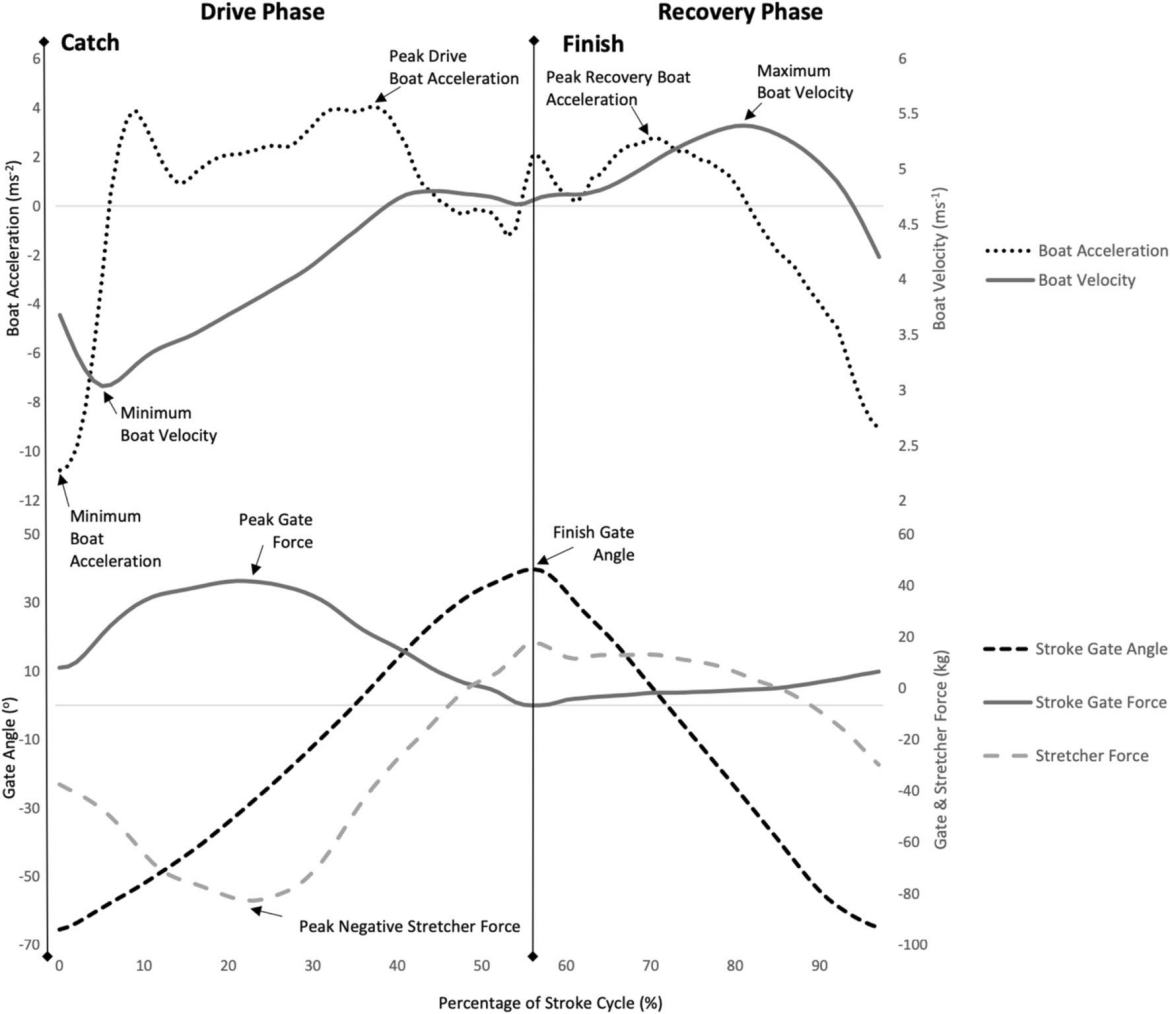
Temporal Profiles per stroke – Boat Acceleration, Boat Velocity, Gate Angle, Gate Force & Stretcher Force

Boat velocity is the key performance indicator in rowing and was reported in 23 of the 27 studies. The positions within a stroke where minimum and maximum velocity occurred [28, 29] and timing from the catch to minimum velocity [30] were also of interest. To provide context to the phases of the stroke cycle, Fig. 4 presents a representative temporal boat velocity per stroke cycle [5]. Fluctuations in boat velocity and velocity range have been discussed in reference to performance [5, 30, 31]. Further, there were other metrics using boat velocity as the outcome comparator. For example, in sweep rowing, the oarside arm was compared to the non-oarside arm in terms of contribution to boat propulsion via measurements of gate force, foot force, power and boat velocity [32] and variations in foot stretcher height were also compared observing the effect on boat velocity [33].

Handle velocity has been measured based on the angle of the oar shaft sensor or gate angle sensor depending on the instrumentation system [34, 35]. Maximal handle velocity during the drive phase has been associated with boat velocity, assuming the blade was completely submerged in the water. A higher handle velocity during the drive phase leads to greater boat acceleration and therefore is positively associated with boat velocity [35].

Seat velocity has been examined in combination with other segment velocities of the handle and trunk and related to the effects on boat acceleration and boat velocity [28] using custom instrumentation systems that included a drum and reel transducer as described by Kleshnev [36] and Draper [37]. Body segment velocities of the legs, trunk and arms were included in two studies as part of calculating the acceleration of the rower’s centre of mass (CM). Through using the CM acceleration, one of these studies described the temporal phases of the stroke cycle through accelerations of the boat and rower [38] while the second study used the rower’s CM acceleration in relation to the determination of mechanical power output [39].

#### Acceleration

Boat acceleration was reported in 8 studies [5, 18, 28, 33, 35, 38–40]. This variable is known to be used in applied sport science settings as a method of technical analysis, but this is yet to be reflected in the peer-reviewed literature [40]. Specific metrics of boat acceleration reported include maximum negative drive acceleration [40], 1st and 2nd peak during the drive [40], time to positive acceleration from the catch [18], time to peak acceleration from the catch, the first dip after the catch, the finish dip [38], and the zero acceleration point before and after the catch [28]. Jerk quantifies the rate at which the boat’s acceleration changes and is measured in m.s^−3^. Six measures of jerk have been reported between the peaks and troughs within a stroke [40]. Furthermore, specific features of the temporal profiles have been described by Kleshnev [38] as microphases within the stroke cycle. This detailed examination delineates five specific micro-phases during the drive, the propulsive segment of the stroke cycle, and three micro-phases throughout the recovery when the rower prepares for the subsequent stroke. These micro-phases demarcate critical transition points where acceleration interchange or momentum shift between the rower and the boat occurs, highlighting moments of potential kinematic and kinetic optimisations. Figure 4 displays the boat acceleration pattern for one stroke cycle.

#### Stroke Rate

Stroke rate was reported in all except one study, ranging from 20 to 41spm. Lower stroke rates were incorporated when other aspects of the rowing stroke were being assessed such as changes to the foot stretcher height or comparing contributions from the oarside and non-oarside arm in sweep rowing [32, 41]. Other studies reported a range of stroke rates and examined how certain metrics changed with higher stroke rate, including shortening of the recovery phase [25, 42]. Although stroke rate was a common metric, it was not uniformly treated as a primary research variable instead it was often included as a parameter in study methodologies.

#### Stroke Length

Stroke length was a focal point of investigation reported in 18 of the 27 studies, with 17 of those examining stroke length in association with the catch and finish angles of the rowing stroke. Measures of stroke length included total angle and effective angle calculated either inclusive or exclusive of the catch and finish slips respectively [25]. The catch and finish slips were quantified by the angular distance covered when the gate force was diminished, falling below a predefined gate force threshold of 196N for the catch and 96N for the finish for sculling [30], with these measurements and thresholds captured using the Peach PowerLine customised software (Peach Innovations, UK). It is important to note the variability arising from the different methodologies utilised to measure these angles. Gate angle calculations were independent of the oar shaft's positioning and were assessed using sensors integrated within the oarlock [30]. In contrast, oar angle measurements were obtained through a potentiometer affixed directly to the oar shaft to register its movements across all three axes [43].

#### Force

Gate force has emerged as a prevalent focus in on-water biomechanical rowing research, reflecting its important influence on performance outcomes [17]. In this current review, 19 force-related metrics were identified. Forces were reported in two planes: horizontal and vertical [5]. Key attributes of force throughout the rowing cycle were considered such as peak force, mean force, rate of force development, mean to peak force ratio and stroke smoothness [5] with Fig. 4 providing a visualisation of the temporal patterns of gate force and stretcher force across the stroke cycle. In addition, some variables, such as peak force, were further considered in terms of where they occur during the stroke cycle and were examined in terms of gate angle position or as a percentage of the cycle at which the peak force was achieved [17]. Further, the contrasting forces exerted by the inside and outside hands on the oar handle were compared [32]. Similarly, in sculling studies, the stroke side (rower’s right-hand side) and bow side force (rower’s left-hand side) profiles were compared for symmetry and the subsequent contribution to boat velocity and boat movements, each of which are elements vital to technical performance optimization [17, 44].

**Fig. 5:**
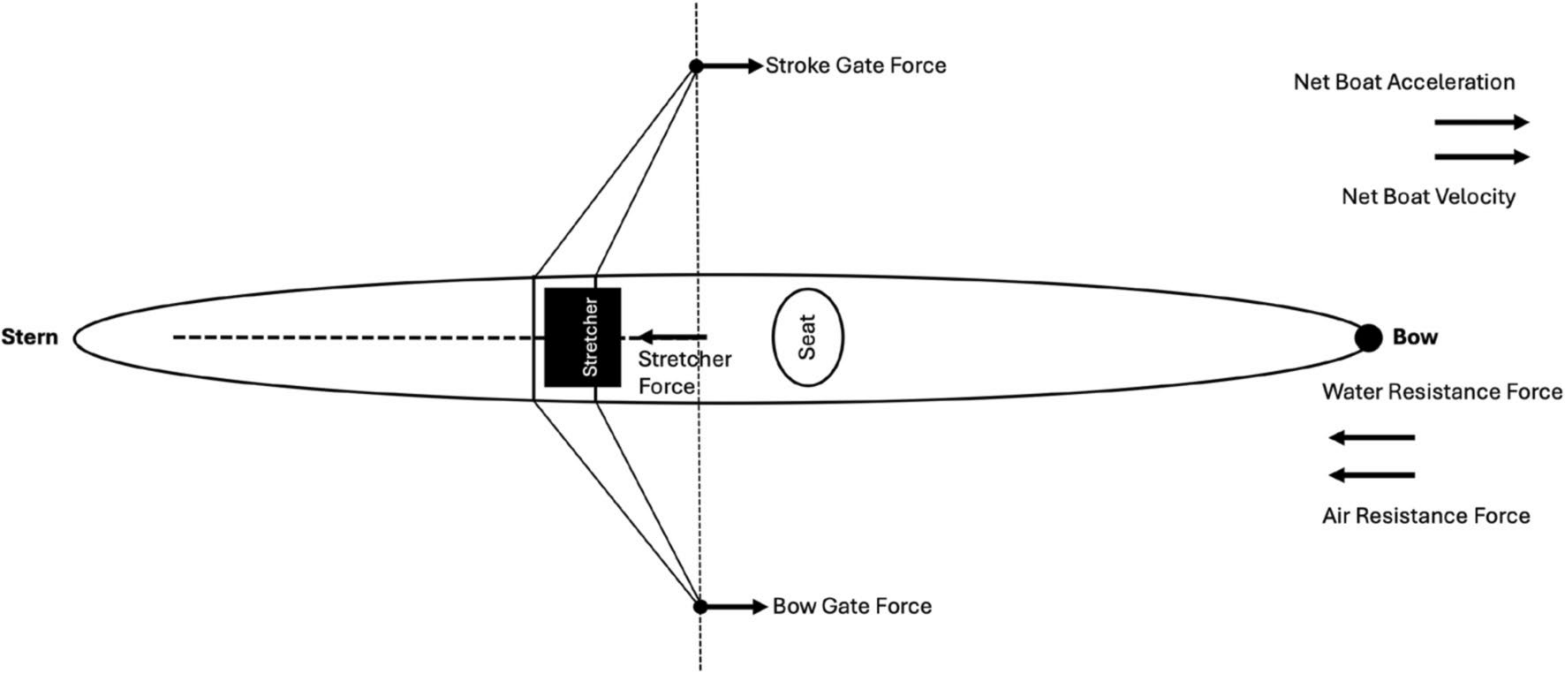
Gate & Stretcher Forces in a Single Scull

Foot stretcher forces, which also play an essential role in contributing to the overall boat propulsion, were measured and reported in 3 of the 27 studies included in this review. Smith and Loschner [5] incorporated foot stretcher force along with gate force to explore the net applied boat force, interpreting its relationship with boat acceleration, and how it affected boat speed in two case studies using the coxless pair and single scull. Net applied boat force plays a crucial role in contributing to the overall boat propulsion and was reported in one study included in this review [5]. The concept of net applied boat force is extensively valued as it captures the real-time interplay of multivariate forces acting on the boat. The net applied boat force is the result of the propulsive pin and foot stretcher forces along with air and water resistance and displays the continuous interaction of the two major opposite-acting forces during the entire stroke cycle (see Fig. 5) [5]. The two other studies that investigated the foot stretcher force were in relation to sweep rowing; specifically the asymmetric patterns of the oarside and non-oarside arm pull and the effect on stretcher force application [32] along with the asymmetrical patterns evident in the stretcher forces during coxed eight rowing in junior rowers [34].

#### Power

Power measurements were reported in 14 of the 27 studies included in this review. The studies detailed both average and maximal power outputs per stroke, alongside relative power metrics normalised for bodyweight, which is particularly important in on-water rowing due to varying athlete stature and body mass. Maximum handle power has shown a strong association with boat velocity, underlining its significance as a performance indicator [35]. Temporal patterns of power output have not been reported in the same way as velocity, acceleration and force measurements and accordingly limited intra-stroke discrete metrics have been explored in on-water rowing. In contrast, mechanical power has been discussed based on two different theories; the common proxy and the true averaged power method [39]. According to Lintmeijer et al. [39] the common proxy method estimates on-water power output as the “time average of the dot product of the moment of the handle force relative to the oar pin and oar angular velocity” (Lintmeijer et al., 2018, p.2138). The true averaged power output also incorporates a residual power related to the mass of the rower, CM acceleration and boat velocity [39].

#### Crew Synchrony

Crew synchrony was reported in 5 of the 27 studies with calculated metrics focusing on the precision, consistency, and coordination of crew movements. Coordination and synchrony in sculling crews was assessed through measurements of boat rotation quantified by pitch, roll, and yaw angles [5] as well as an examination of translational boat movements, including surge, heave, and sway [45]. Synchronisation in coxless fours was assessed through detailed analysis of force curve profiles whereby timing differences at the onset and finish of the stroke were considered a synchronisation indicator [46]. In addition, a range of variables were reported such as differences in the area under the force curve and form differences examined in the force curve profiles, where the individual’s force curve pattern was presented as a percentage difference of the average force curve for the crew [46]. Baudouin and Hawkins [47] hypothesized that crew performance could be predicted from total propulsive power, level of synchronisation and total rower drag contribution. Timing differences and the adaptability of the force curve profile that occurred with changes to rower combinations were observed. Specifically, comparisons of crew synchrony were assessed through the interpretation of propulsive blade force profiles where rowers demonstrated the ability to adapt their biomechanics appropriately based on the feedback within the rowing system or crew after only a brief period of time [47]. Likewise, the coordination and consistency of the bow four rowers in a coxed eight were assessed through the force–time profiles. The average and variability of force–time profiles were determined to characterise the patterns of variation in maximum force, stroke duration and inter-stroke interval [48]. Such detailed assessment of force–time traits is essential to interpret the complexities of crew synchrony and its impact on collective rowing efficiency.

## Discussion

This scoping review aimed to describe the current scope and density in the field of on-water rowing biomechanics and provide a guide for practitioners and researchers on future directions for the advancement of biomechanical studies in on-water rowing. Measurement systems, study characteristics and reported biomechanical variables were collated to describe the state of the on-water rowing literature and to provide a guide for future directions for rowing biomechanics research. Data extraction revealed stroke rate, gate force, horizontal oar angle and boat velocity as the most reported variables with numerous subcategories of metrics within each measure. Boat acceleration has been the focus of less research in comparison to force and velocity, but has the potential to provide further insights as an important boat outcome measure.

### Study Characteristics

The majority of the included studies in this scoping review were observational in design. Further, given the logistical and environmental considerations of on-water rowing assessment, the majority of the studies included in this review were cross-sectional. The criterion of on-water rowing assessment for this review reduced the number of relevant research studies, with 18 rowing ergometer studies excluded during the screening process for utilising the rowing ergometer as the assessment platform. These studies involved some aspect of biomechanical assessment related to rowing; however, the outcomes of these studies cannot definitively be extrapolated to on-water rowing outcomes due to the recognised technical differences between ergometer and on-water rowing [49–52]. Many of the participants in the included research were from elite or sub-elite populations which often leads to small sample sizes; however, such populations elicit a high level of ecological validity. To increase sample size and statistical power, along with the applicability of the findings towards youth, masters and developmental pathways, larger demographic groups could be examined in future research to expand the scope of investigation, including club, collegiate and masters rowing populations.

### Biomechanical Variables

#### Boat Velocity

Boat velocity and 2000 m race time are generally considered to be the most fundamental performance outcomes in on-water rowing [6] and expectedly boat velocity was one of the most reported outcome measures across the studies in this review. Boat velocity can be a difficult parameter to compare between studies due to the variability of the environmental conditions [7]. With an increase in water temperature, boat velocity can increase significantly [53]. Therefore, it is common to use time margins and 500 m time splits for comparison across races, competitions and venues [54]. Average boat velocity can also be misleading as a measure; however discrete metrics including minimum, maximum, range and fluctuations in velocity can provide a more detailed appraisal of performance [28]. Intra-stroke fluctuations in boat velocity have been discussed in reference to performance. Further, intra-stroke velocity fluctuation relates to the interaction between the drive and recovery phases and the efficiency of the rower to maximise their average boat velocity while minimising disruptions to the boat run [13]. A reduction in velocity fluctuations will likely lead to superior average boat velocity and subsequently enhanced performance [5, 30, 31]. Exploring the velocity profile pattern of a rower or crew has the potential to provide deeper insight and highlight different technical strategies, particularly when average boat velocity is similar between crews [28]. Subsequent analysis of other variables, such as acceleration, force or body segment coordination may assist in the explanation of variance in crews’ technical strategy [40]. All of these metrics need to be considered to understand the efficiency of the rower and their individual approach and technical strategy.

#### Handle and Seat Velocity

Handle velocity during the drive phase has a strong positive association with boat velocity [35] and during the drive phase increases with faster stroke rate [34]. Furthermore, handle velocity is reduced with increased gearing ratios unless handle force is elevated to maintain handle velocity with the higher gearing [42]. Seat velocity was reported alongside body segment velocities at the handle and trunk, representative of the three main body segment movements during the stroke cycle: leg drive, trunk swing and arm draw [38]. When comparing between stroke rates, the handle, seat and trunk velocity measures are not different when stroke rate increases. In contrast, the recovery velocity of legs, trunk and arms increases significantly with increasing stroke rate, and this occurs across all boat categories [24]. The on-water rowing research lacks information about body segment coordination and it is not known which rowing style is the most effective at generating gate force and boat propulsion [55]. This area of rowing biomechanics has largely been explored using rowing ergometers in laboratory settings where access to motion capture equipment is readily available. Further, a large proportion of rowing literature utilises the rowing ergometer rather than on-water rowing due to the convenience and environmental stability of the laboratory. In addition, some aspects of biomechanics research require the use of equipment that is not available in the mobile aquatic environment such as motion capture systems that facilitate the biomechanical assessment of body segment and joint position tracking. Markerless motion capture systems are emerging and have the potential to assess on-water rowing kinematics; but they have not been validated in the on-water rowing environment [56]. Therefore, there is currently no equivalent substitute for three-dimensional motion capture in the on-water environment; however, sensor technology is quickly gaining traction and may be applicable to on-water rowing movement assessment in the near future [16, 57].

Assumptions can be made about rowing technique through the observation of seat velocity during the drive phase as it signifies the beginning and end of the leg drive, revealing how rowers coordinate their power output [5]. A comparison between lightweight and heavyweight male coxless pair crews exhibited similar boat velocity even though the heavyweight crew displayed higher force and work outputs, suggesting different technical strategies enable the lightweight crew to efficiently achieve equivalent boat velocity [28]. Future studies investigating the combination of seat velocity, handle velocity and trunk velocity have the potential to better understand body segment coordination and the technical strategies that affect performance outcomes such as boat velocity and boat acceleration.

#### Acceleration

Boat acceleration is measured per stroke and several intra-stroke metrics have been identified in on-water rowing [38, 40]. Boat acceleration metrics that have been associated with superior performance outcomes or greater boat velocity have been primarily focussed within the drive phase of the stroke cycle with a particular focus ranging from the catch to peak acceleration. The catch and initiation of force application during this propulsive phase are critical aspects of the rowing stroke cycle; however key areas through the finish and recovery phase have the potential to inform and improve technique [40]. The finish signifies the beginning of the recovery, and the rower is executing a technical movement pattern to compress the body throughout the recovery without disrupting the boat run whilst maintaining boat velocity to prepare for the next catch and drive phase. The conservation of momentum and inertia is vital to maintaining boat velocity that was generated earlier in the drive phase. Figure 4 displays an example of typical force, acceleration and velocity profiles for one stroke cycle, highlighting the catch, drive, finish and recovery sections of the stroke.

Boat acceleration is an outcome measure in rowing biomechanics and provides a reflection of the force applied at both the gate and foot stretcher, often referred to as the applied net boat force [5]. Discrete metrics of boat acceleration have been reported in the literature; however, some conflicting results have made the interpretation of optimal profiles challenging [38, 40, 58]. The gate angle at peak acceleration has been identified as a variable that could distinguish between different levels of rower [35], with an earlier peak force in the stroke cycle related to superior performance. If the force output can be maintained through to the blade release, a sustained force will provide a higher mean force along with greater mechanical work done and subsequently sustain boat velocity [59]. Olympic champion level rowers displayed a deeper negative acceleration peak around the catch when compared to national level rowers [5] and based on the assumption this is due to a faster leg drive, this was associated with superior performance outcomes [33]. Moreover, foot stretcher height has been investigated through known metrics including boat acceleration to optimise performance and a higher foot stretcher height increased the negative acceleration peak around the catch [33]. This may potentially be attributed to the magnitude and direction of the foot stretcher force being applied with a higher foot stretcher height, but the stretcher force was not directly measured in this study by Liu et al. [33].

Changes in jerk (rate of change of acceleration), measured in single sculls and coxless pairs over 2000 m races have been considered in relation to boat velocity. Greater absolute values of jerk in the early drive, mid-drive and late recovery were associated with superior performance outcomes across a sample of single scull and coxless pair crews [40]. Along with jerk, time to positive acceleration distinguished between the perception of ‘good’ and ‘bad’ strokes using rowers’ performance-based judgements [18]. In addition to the assessment of the discrete metrics, the characteristic shape of the boat acceleration pattern per stroke represents the outcome of an individual’s technique, and it therefore has the potential to provide objective feedback in the on-water daily training and competition environment [38, 60]. Research utilising functional data analysis to assess the temporal force curve patterns in on-water rowing [61] can be applied to the temporal pattern of boat acceleration to further understand the idiosyncrasies of individual signature profiles [62]. Further research is warranted utilising higher dimensional statistical approaches such as functional data analysis with the potential to explore time series analysis of temporal patterns of biomechanical rowing variables such as velocity, acceleration and force to better understand technical strategies related to performance [59].

#### Stroke Rate

All studies reported stroke rate with the exception of one study [48]; however the stroke rate was often reported in the methodology as a procedural requirement and was not part of the research question. Stroke rate can vary in range depending on prescribed intensities in training or race conditions on the day of competition. Further, reporting of stroke rate differs between studies, making comparison challenging. The majority of on-water rowing training is completed at relatively lower stroke rates [12], but the application of force, power, and the management of momentum of the rower-oar-boat system is markedly different when rating 20 spm compared to 40 spm [63]. Stroke rate during Olympic final races ranges from an average of 34 spm in the women’s single scull event up to an average of 40 spm for the men’s eight event (BioRow, 2024). The prescribed stroke rate chosen for a research study should best reflect the research question. For example, if the purpose of the study is to assess an aspect of performance, race rating and race conditions would be optimal. However, given a large proportion of on-water rowing training is completed at lower stroke rates [12], research questions may specify a lower stroke rate or range of stroke rates for assessment.

#### Stroke Length

Stroke length was reported in 18 out of 27 of the studies included in this review. The choice of angle measurement technique in on-water rowing can substantially influence the recorded stroke length data, emphasizing the importance of standardization of methods across studies to enable meaningful comparisons. For example, the predefined gate force threshold used to calculate the catch and finish slips are applied the same across all sexes, ages and weight classes. This may be a limitation given the peak forces are different across these different demographic groups [27, 30]. This methodological distinction is of paramount importance for interpreting biomechanical data, as it may influence the perceived effectiveness of each rowing stroke.

A longer stroke length reportedly relates to superior performance, resulting in greater average boat velocity [51], as it provides a longer drive distance to generate force on the gate. Stroke length directly affects stroke rate; however, stroke length has been shown to remain stable in stroke rate ranges from 20 to 28 spm [25]. However, at 36–40 spm, a relatively high range of stroke rate, the stroke length may decrease by 3–4 degrees in sweep rowing and 5–6 degrees in sculling [25]. Stroke length varies depending on sculling or sweep rowing, boat category, weight category and athlete demographics. From the studies in this review stroke length ranged from 78 to 88 degrees for sweep rowing and 100–106 degrees for sculling. Effective angle, which excludes catch and finish slip angles from total stroke length was unable to discriminate between elite and sub-elite rowers under race conditions [25]. However, the finish slip was identified as the most discriminating feature between a group of world-class female rowers [35]. Further, catch and finish slips, highlight the degree of gate angle where the force applied does not reach a pre-determined threshold and does not contribute to boat propulsion or influence boat velocity [30].

Reporting the percentage of stroke length for certain discrete metrics is also common in this domain. For example, the angle at peak force is a commonly reported measurement and earlier peak force has been associated with superior performance outcomes [30]. In addition, if peak force is achieved earlier and maintained longer, this results in a greater mean force per stroke which is also associated with higher performance [61]. However, greater mean force per stroke does not necessarily translate to a faster boat velocity [14, 28] and further consideration of other variables is required to understand the technical efficiency and strategy of a crew.

#### Force

Gate force or handle force in the on-water rowing literature has generated considerable attention and inquiry over the last 5 decades given its direct connection to boat propulsion and performance [17]. Assessment of temporal force profiles has been extensively explored alongside discrete metrics of force, including peak, mean, time to peak, mean to peak ratio and rate of development. The catch and finish force gradients reflect how quickly the rower applies the force after the catch and how long they can maintain the force at the back end of the stroke leading towards the finish based on a predetermined threshold of 30% of peak force at either end of the drive phase [25]. The ability to maintain force for longer into the finish of the stroke was a distinguishing feature of elite rowers when compared to sub-elite [25] and practitioners could use this information when planning training drills around specific elements of the stroke.

Vertical force is measurable at the gate, handle, and foot stretcher dependent on the instrumentation system. Vertical gate force is influenced by the pitch of the oar blade [5] and is important when considering the non-propulsive forces on the boat and subsequent effects on propulsive boat acceleration, velocity and movement [5]. Multi-axial forces are measured in rowing biomechanics; however, one or two dimensions are most commonly reported [5, 17]. In addition, force can be measured as propulsive [5] or the normal component [38]. Therefore, when making comparisons of forces between studies, it is essential to clarify the method used to measure the force.

The addition of foot stretcher instrumentation adds complexity to the measuring system, reducing portability, increasing set up time and is therefore a less common inclusion in on-water rowing studies [5]. However, along with drag and water resistance, foot stretcher forces are an important component in the applied net boat force [5] and this variable relates to the boat acceleration when comparing temporal profiles across the stroke cycle [37]. The temporal pattern of the propulsive net applied boat force features the qualitative differences between rowers’ individual technique and also reflects the boat propulsion. In sweep rowing, a characteristic asymmetry of the stretcher force has been thought to be caused by the rotation of the sweep oar around the pin followed by the rower’s movement through the stroke cycle [34]. With these findings in mind, future research should incorporate foot stretcher force as applied net boat force incorporates gate force and foot stretcher force to provide a more detailed picture of the propulsive forces acting on the boat [59].

#### Power

The accuracy of quantifying mechanical power in on-water rowing is important to gauge and predict performance [39]. Two methods have been reported in the reviewed studies: the common proxy method and the averaged true power method [36, 64]. As with a multitude of sporting activities involving propulsion, power is lost within the rowing cycle, as the boat does not travel at a constant velocity [65]. The mechanical power lost to drag is proportionally greater at higher velocities due to the exponential relationship between drag and velocity. A proportion of the net mechanical power is used to overcome the resistance caused by the velocity fluctuations within each stroke cycle and can be quantified in terms of the velocity efficiency estimated to be around 5–10% of the net mechanical power [26, 65].

Power per kilogram of body weight in relation to boat speed has been investigated to compare heavyweight and lightweight men’s coxless pair crews [14]. The heavyweight crews consistently achieved significantly higher Power at five different stroke rates varying from 20 spm up to race rate. However, the higher peak and average handle forces elicited by the heavyweight rowers were not reflected in the boat velocities, with two lightweight crews exhibiting equivalent boat velocities to the heavyweight crews. It was evident that lightweight crews were potentially able to perform to a similar level by adopting more effective technical strategies [14]. This information may inform the development of race tactics or squad selection strategies.

Power application from a technical perspective in on-water rowing should also be prioritised along with force application and boat velocity [30]. The mean power needed to achieve a race performance level, can be used as a target in setting training strategies and prescription [31]. The research has directed attention toward understanding how stroke rate influences net mechanical power [40, 64] along with the subsequent effects on boat acceleration and boat velocity [26, 40]. These findings are instrumental for understanding the complex interplay between rowing technique, power application, and resultant performance, revealing avenues for targeted enhancements in competitive rowing. However, the relationship between rowing power output and stroke rate, gearing and drag factor has been reported with results suggesting there is no optimum relationship with stroke rate, or gearing to elicit maximum power in rowing [42]. This was in contrast to other sports such as cycling and swimming, where an optimal trend has been reported [42]. In swimming, velocity decreases if stroke rate exceeds a certain value [66] and in cycling, specific power outputs can be linked to an optimal cadence which is linked to muscle activation efficiency [67]. There is an absence of conclusive literature in this area and there is likely a complex and dynamic combination of factors that influence optimal stroke rate for an individual or crew. Further investigations are needed to ascertain the importance and relevance of the determination of an optimal stroke rate in on-water rowing.

Finally, Holt et al. [30] investigated measures of rowing technique and performance and their relationship with boat velocity, taking into consideration differences in boat classes and sex. Improving the force generating capacity of the rower was recommended as a key component for power output in the pursuit of rowing performance improvement [30]. Moreover, a degree of asymmetry of the stretcher force is necessary in sweep rowing for a high-power output, but excessive foot stretcher asymmetry may lead to an increased risk of overloading the lumbar spine due to shear forces, with no optimal range specified [34]. It is clear that power is a critical measure to incorporate into monitoring and controlling training loads for rowing and this has been extensively studied on the rowing ergometer and in relation to strength training and assessment for rowing [68, 69]. However, further investigations may improve our understanding on power application during on-water rowing to optimise performance.

#### Crew Synchrony

For a rowing crew to be successful, a high level of coordination and synchrony between crew members is required to achieve optimal performance [46]. Crew synchrony can be defined as the simultaneous actions of all crew members and is essential in crew rowing in relation to detrimental boat movements and lateral stability [45]. Cuijpers et al. [45] demonstrated that crew coordination was more consistent with increased stroke rate and superior crew synchronisation. However, fluctuations in boat movements including surge (forward-linear motion), heave (vertical-linear motion) and pitch (lateral axis rotation) increased while lateral movements measured as roll (long axis rotation) decreased. These results suggest superior crew synchronisation may relate to enhanced lateral stability. However, this inevitably involves lower biomechanical efficiency due to the increased surge, heave and pitch [45]. These boat movement patterns were largely due to the cyclical and fluctuating nature of the rowing stroke cycle, where heightened coordination can potentially lead to greater power production as a crew [45]. Boat movements including pitch, roll and yaw were only explored in relation to crew synchronization [45]; however, excessive additional boat movement and rotation negatively affects boat propulsion and reflects the technical efficiency of a crew or rower [70]. It is clear that this area of inquiry is in its infancy and more research can be undertaken in this area to inform practice.

The literature pertaining to on-water rowing synchrony included in this review reveals a focus on small boat categories to assess the individual contribution to the boat output rather than the crew performance. From a research perspective it is important to improve our understanding of the biomechanical factors associated with successful technique and enhanced performance. However, the synchrony within a crew, the selection of a crew and the most appropriate seating order within a crew to achieve success are also relevant research questions, particularly given the coxed eight is often considered the most prestigious event in the regatta schedule [71]. Coaches seeking to optimise crew selection can also consider the suitability of individual rowers in a crew through the adaptability of a rower’s force–time profiles to increase the level of synchrony and how that affects boat movement and performance outcomes [47].

### Summary

This scoping review has identified a range of biomechanical variables that have been assessed during on-water rowing and presents a myriad of applications of these attributes in relation to rowing performance. The average boat velocity over a measured distance or interval, in racing or training is considered a fundamental performance outcome together with race time. Intra-stroke metrics of interest for boat velocity were minimum, maximum and range measurements [26]. Velocity has also been measured at the handle and the seat, in relation to gearing and body segment movements respectively. Power has been reported in absolute and relative measures of maximum and average power per stroke and measured at the gate and handle [42, 62]. Force measured at the gate, handle or oar has received a large degree of research attention given its relationship to boat propulsion [59]. The temporal gate or handle force pattern has been extensively dissected and descriptively characterised over many decades and discrete force metrics of interest include peak force, mean force, mean to peak force ratio, gate angle or time to peak force and catch and finish gradients of force [31]. The ability to achieve a rapid rate of force development early in the drive as well as maintaining that force for longer into the finish are considered distinguishing features of successful on-water rowing performance [25, 30]. Stretcher force was a less common inclusion in the literature due to increased complexity of the instrumentation system set up. However, the combination of gate force and stretcher force measurements facilitates the assessment of net boat force which offers a more comprehensive assessment of the propulsive forces acting on the boat and can be related to the boat acceleration temporal profile [5].

Discrete metrics of boat acceleration reportedly relate to changes in acceleration between the boat and rower and may be associated with individual technique characteristics and performance outcomes [38]. Peaks and slumps have been identified in the boat acceleration during the drive and recovery phase that relate to certain points during the stroke cycle [40], yielding implications for training design for coaches and performance analysts. Moreover, jerk has been associated with performance based on the impact to the boat velocity [40]; however, additional research is required to more thoroughly investigate the discrete metrics and temporal profiles of boat acceleration in relation to performance and rowing technique to establish conclusive recommendations. The measurement of boat acceleration is non-invasive and requires no adjustment to the boat or rigging set up; it therefore has the potential to provide the athlete, coach and support staff with objective feedback in the daily training and competition environment. The cost effectiveness of inertial sensors and the availability of relevant software in smart devices, makes boat acceleration an accessible metric for all levels of the sport. Further, the measurement of boat acceleration encompasses the drive and recovery phases of the stroke, making it a suitable measurement tool for on-water technique assessment.

The recovery phase of the stroke cycle is perceived by coaches to require a high level of skill including balance, coordination, rhythm and feel for the boat run [13] as the oars are out of the water and minimal mechanical work is occurring during this time. This phase is concerned with managing the momentum that has been gained during the drive phase and it is clear that further understanding of the recovery phase and its contribution to maintaining boat speed throughout the stroke cycle is required. Conceivably, the on-water metric of distance per stroke provides an all-encompassing measure of both the drive and recovery phases, given it decreases with an increased stroke rate; however, the sequencing of body segments from finish to catch and the effect on the boat velocity and acceleration during this time may provide further insights. Additional research is required into the assessment of body segment coordination and joint position in the on-water rowing environment as the evidence from the rowing ergometer literature does not reflect entirely what is occurring in on-water rowing. Investigations examining coordination of the three main body segments alongside identifying joint motion in the hips, ankles, trunk and shoulders with boat outcome measures could provide valuable understandings on the mechanisms responsible in relation to the most effective rowing technique. Moreover, rowing technique and biomechanical variables assessed at regular intervals over an extended period of time involving the same participants has the potential to demonstrate the extent to which some technical changes are possible and can be measured and monitored through an individual’s temporal profiles of force, acceleration and velocity.

In summary, the literature has reported on an extensive range of biomechanical metrics encompassing time, space and force that are relevant to rowing performance. The variability of reported measures throughout the different boat classes, sex and skill levels makes the collation of data challenging. However, establishing a guide may provide recommendations to standardise the description of variable names, assessment methods and on-water testing protocols. This could assist to advance on-water rowing biomechanical assessment so that systematic reviews and meta-analyses in the future can provide robust conclusive statements on biomechanical factors and their association with rowing technique and performance.

## Conclusion

This is the first scoping review of the on-water rowing biomechanics literature, with the search including all peer-reviewed papers published until 29 September 2023. The results provide an overview of the extent of peer-reviewed knowledge in on-water rowing biomechanics measurement and associations with performance. The review also provides an overview of the participant characteristics and range of variables reported in the on-water rowing literature. Rowing biomechanics research has additional layers of complexity given there are two types of rowing: sculling and sweep rowing, two categories of rowers, lightweight and heavyweight, as well as multiple boat categories involving one person in a single scull and up to eight people in a coxed eight. This makes the collation of results across the body of literature into a succinct summary challenging. The single scull and coxless pair were the most common boat categories for research studies, unsurprisingly, given they are the small boat categories that best represent the individual output on the boat. The coxless four and double scull were underrepresented while the quadruple scull was not represented in the research at all.

On-water rowing assessment has well-established parameters for the interpretation of force profiles, with discrete and temporal analyses applied to sculling and sweep rowing studies. The rate at which a rower can apply force and the ability to maintain the force into the finish are distinguishing features of elite rowing. In on-water rowing, prioritizing the measurement and application of power is essential for effectively monitoring and controlling training loads, as well as for refining technique. Boat acceleration is considered a reflection of the applied net boat force; however higher dimensional statistical approaches such as functional data analysis should be explored to understand the temporal differences in boat acceleration that lead to superior performance. Ultimately, the development of a standardized framework for on-water rowing biomechanical assessment, coupled with established protocols for environmental data collection, would provide practitioners and researchers with a structured approach for navigating the on-water rowing context. The standardisation of an on-water testing protocol to include a range of stroke rates and distances, depending on the research question, may assist in future collation of original rowing research. Furthermore, the development of guiding principles on reporting the specifications of instrumentation systems, sampling rates and sensor locations may assist with the standardisation of methodologies and facilitate more direct comparison across studies. The implementation of such standardisation has the potential to foster increased research that employs on-water assessment techniques, thereby deepening the understanding of the technical intricacies and performance metrics unique to the sport of rowing.

## Data Availability

Not applicable.

## Abbreviations

CM: Centre of mass
F: Female
HW: Heavyweight rowers
kg: Kilogram
LW: Lightweight rowers
M: Male
m: Metre
m.s^−^^1^: Metres per second
m.s^−^^2^: Metres per second squared
m.s^−^^3^: Metres per second cubed
N: Newton
NZ: New Zealand
PCC: Population concept context framework
PRISMA-ScR: Preferred reporting items for systematic reviews and meta-analyses extension for scoping reviews
spm: Strokes per minute
UK: United Kingdom
USA: United States of America
v^3^: Velocity cubed
1x: Single scull
2x: Double scull
2-: Coxless pair
2D: Two-dimensional
4x: Quadruple scull
4-: Coxless four
8+: Coxed eight
%: Percentage
°: Degrees
